# Partial Nitrification and Enhanced Biological Phosphorus Removal in a Sequencing Batch Reactor Treating High-Strength Wastewater

**DOI:** 10.3390/ijerph19095653

**Published:** 2022-05-06

**Authors:** Xiaojun Feng, Yishi Qian, Peng Xi, Rui Cao, Lu Qin, Shengwei Zhang, Guodong Chai, Mengbo Huang, Kailong Li, Yi Xiao, Lin Xie, Yuxin Song, Dongqi Wang

**Affiliations:** 1 Xi’an Modern Chemistry Research Institute, Xi’an 710065, China; xiaojunfeng204@outlook.com (X.F.); qys1017@stu.xjtu.edu.cn (Y.Q.); pengxi204@outlook.com (P.X.); 2 Department of Environmental Science and Engineering, School of Energy and Power Engineering, Xi’an Jiaotong University, Xi’an 710049, China; 3 Department of Municipal and Environmental Engineering, Xi’an University of Technology, Xi’an 710048, China; 2180420032@xtu.xaut.edu.cn (R.C.); 1170411051@xtu.xaut.edu.cn (L.Q.); 2190421301@xtu.xaut.edu.cn (S.Z.); 1190411045@xtu.xaut.edu.cn (G.C.); 2210421298@xtu.xaut.edu.cn (M.H.); 2200421171@xtu.xaut.edu.cn (K.L.); 2200421173@xtu.xaut.edu.cn (Y.X.); 2200421182@xtu.xaut.edu.cn (L.X.); 2210420004@xtu.xaut.edu.cn (Y.S.); 4 State Key Laboratory of Eco-Hydraulics in Northwest Arid Region, Xi’an University of Technology, Xi’an 710048, China; 5 Shaanxi Key Laboratory of Water Resources and Environment, Xi’an University of Technology, Xi’an 710048, China

**Keywords:** high-strength wastewater, partial nitrification, enhanced biological phosphorus removal, polyphosphate accumulating organisms, nitrifying bacteria

## Abstract

Complex and high levels of various pollutants in high-strength wastewaters hinder efficient and stable biological nutrient removal. In this study, the changes in pollutant removal performance and microbial community structure in a laboratory-scale anaerobic/aerobic sequencing batch reactor (SBR) treating simulated pre-fermented high-strength wastewater were investigated under different influent loading conditions. The results showed that when the influent chemical oxygen demand (COD), total nitrogen (TN), and orthophosphate (PO_4_^3−^-P) concentrations in the SBR increased to 983, 56, and 20 mg/L, respectively, the COD removal efficiency was maintained above 85%, the TN removal efficiency was 64.5%, and the PO_4_^3−^-P removal efficiency increased from 78.3% to 97.5%. Partial nitrification with simultaneous accumulation of ammonia (NH_4_^+^-N) and nitrite (NO_2_^−^-N) was observed, which may be related to the effect of high influent load on ammonia- and nitrite-oxidising bacteria. The biological phosphorus removal activity was higher when propionate was used as the carbon source instead of acetate. The relative abundance of glycogen accumulating organisms (GAOs) increased significantly with the increase in organic load, while *Tetrasphaera* was the consistently dominant polyphosphate accumulating organism (PAO) in the reactor. Under high organic loading conditions, there was no significant PAO–GAO competition in the reactor, thus the phosphorus removal performance was not affected.

## 1. Introduction

High-strength wastewaters, mainly originating from livestock and poultry farming and food processing, have higher pollutant concentrations and ecological risks than conventional domestic wastewater [1]. The chemical oxygen demand (COD) concentration in high-strength wastewater can be as high as tens of thousands of milligrams per litre, total nitrogen (TN) levels can reach 800–23,000 mg/L, and total phosphorus (TP) levels can reach 50–230 mg/L [2,3,4,5]. If discharged directly into receiving water bodies without treatment, such large amounts of organic matter and nutrients in the wastewater will consume dissolved oxygen (DO) and cause eutrophication, promoting algal growth. This can lead to the death of aquatic organisms and the deterioration of the water environment [6]. Therefore, the treatment of high-strength wastewater is an urgent matter. Anaerobic digestion (AD) is one of the preferred methods, as it can economically and effectively treat highly concentrated organic wastewater, promoting carbon neutrality through energy recovery [7,8,9]. However, AD does not address the problem of excess nitrogen and phosphorus in high-strength wastewater. The removal of phosphorus from wastewater, in particular, is less studied, making the AD effluent still at risk of causing eutrophication in the receiving water body [4,10].

The enhanced biological phosphorus removal (EBPR) process has been widely used to remove phosphorus from domestic wastewater. This process relies on the enrichment of polyphosphate accumulating organisms (PAOs) in activated sludge [11]. Under anaerobic conditions, PAOs assimilate volatile fatty acids (VFAs), such as acetic acid and propionate acid, and break down stored polyphosphates and glycogen to generate energy and reducing power, whereas under aerobic conditions, they take up an excessive amount of phosphorus, thus achieving phosphorus removal from the wastewater [12]. In addition to PAOs, the main microorganisms in the EBPR system are glycogen accumulating organisms (GAOs), which behave similar to PAOs but do not have the ability to store polyphosphate [13]. Therefore, GAOs compete with PAOs for carbon sources during the anaerobic phase of the EBPR system but are unable to remove phosphate from the wastewater. Deterioration of the EBPR system is also attributed to an increase in the abundance of GAOs. Previous studies on EBPR systems have generally focused on optimally tuning the phosphate removal performance under relatively low organic loading conditions to give PAOs a competitive advantage over GAOs [14,15,16,17], while studies on medium-to-high-strength wastewaters containing high concentrations of organic matter (>400 mg/L) and phosphorus have been very limited [18].

EBPR process can be used to treat high-strength wastewater with different influent COD/P ratios (25:1 to 10:1), and the phosphorus removal efficiency could be maintained above 70% [19,20]. The phosphorus removal efficiency in EBPR systems treating wastewater containing high concentrations of phosphorus (30–280 mg P/L), such as dairy and manure wastewater, could be 60–90% [21,22]. However, these studies lacked a comprehensive analysis of the changes in the nitrogen and phosphorus removal performance, microbial activity and community structure in the systems. Previous studies showed that an excessively high influent COD/P ratio (>50:1) in the EBPR process treating low-strength wastewater promotes the proliferation of GAOs, which in turn affects the EBPR performance [12]. Randall and Chapin found that high influent carbon source concentrations (>740 mg COD/L) reduced the phosphorus removal stability and EBPR activity, and attributed to the fact that high organic loads favoured the growth of non-PAO and led to PAO being screened out of the system [23]. However, it is still unknown whether a carbon source competition between PAOs and GAOs occurs in high-strength wastewaters wherein various types of available carbon sources are sufficient. Therefore, it is necessary to evaluate the relationship between different functional microorganisms and the metabolic activity of different carbon sources in the EBPR process treating high-strength wastewater to ensure efficient and stable system performance. 

In this study, a laboratory-scale anaerobic/aerobic sequencing batch reactor (A/O-SBR) was constructed to treat pre-fermented high-strength wastewater. The main objectives of our study were to (1) investigate the pollutant removal performance, microbial activity, and community structure in the reactor under different influent loading conditions; (2) evaluate the impact of organic load on nitrogen and phosphorus removal activity/populations, and (3) reveal whether the carbon source competition among different functionally relevant microorganisms occurs in high-strength wastewater. The outcome will provide support for the design and optimisation of the biological treatment of high-strength wastewater. 

## 2. Materials and Methods

### 2.1. Reactor Setup and Operation

An SBR with a working volume of 4.62 L (Figure S1) was constructed in the laboratory. The seed sludge was taken from the No.4 wastewater treatment plant (WWTP) in Xi’an, Shaanxi Province, washed three times with tap water, and aerated before inoculating in the reactor. The reactor was operated under ambient temperature (20 ± 5 °C) without pH control. The variations in pH in the reactor (median: 7.4; range: 7.0–8.5) were possibly related to the protein hydrolysis during the experiment, yet the values are still in the suitable range for PAO activity [13,24], and the previously reported range for typical livestock wastewater (6.8–8.9) [25]. The cycle time was 8 h (anaerobic: 2.5 h; aerobic: 4.5 h; settling and draining: 1 h), and the sludge retention time (SRT) was ~14 d. In each cycle, 2.41 L of synthetic wastewater was pumped into the SBR, resulting in an exchange ratio of 0.52 and a hydraulic retention time (HRT) of 15.3 h.

The pollutant components and concentrations in the synthetic pre-fermented high-strength wastewater were defined based on the real wastewater from a local livestock farm in Xi’an, Shaanxi Province, which are also within the reported range of high-strength dairy and manure wastewater (Table S1) [26,27,28]. A mixture of complex (casein acid hydrolysate) and simple organic matter (sodium acetate and sodium propionate) in a COD ratio of 2:7:7 was used as the carbon source. Ammonium chloride and monopotassium phosphate were used as the inorganic nitrogen and phosphorus sources, respectively. During the start-up period (60 days), the influent COD (COD_inf_) concentration was gradually increased from ~200 to ~330 mg/L, while the influent ammonia (NH_4_^+^-N_inf_) and orthophosphate (PO_4_^3−^-P_inf_) concentration was ~20 and ~8 mg/L, respectively. During Phases I, II, and III, the COD_inf_ concentrations were ~400, ~700, and ~1000 mg/L; the NH_4_^+^-N_inf_ concentrations were ~20, ~35, and ~50 mg/L; and the PO_4_^3−^-P_inf_ concentrations were ~8, ~15, and ~20 mg/L, respectively, resulting in a COD/N ratio of ~20:1 and a COD/P ratio of ~50:1 (Table 1). The concentrations of other trace elements (Table S2) [29] were kept constant during the experiment. To evaluate the pollutant removal performance during experiments, the influent and effluent concentrations of COD, TN, NH_4_^+^-N, nitrate (NO_3_^−^-N), nitrite (NO_2_^−^-N), TP, and PO_4_^3^^−^-P were regularly monitored. 

### 2.2. Typical Cycle Study and Biological Phosphorus Removal Batch Tests

Samples were taken at different time points during one operating cycle of the reactor to analyse the changes in COD, TN, NH_4_^+^-N, NO_3_^−^-N, NO_2_^−^-N, and PO_4_^3−^-P, in order to determine the pollutant removal kinetics and activities of the reactor during a typical cycle.

To assess the EBPR activity of the sludge, anaerobic/aerobic batch experiments were conducted as described previously [11]. Briefly, sludge samples at the end of the aerobic period in the reactor were taken and washed three times with a washing solution without carbon and phosphorus sources [29]. Allyl-N-thiourea (ATU) was added into the mixed liquor to inhibit nitrification, and the air was pumped in for 1 h of pre-aeration [30]. Then, after the residual organic matter had been consumed, nitrogen gas was pumped in to bring down the DO level to <0.1 mg/L to attain anaerobic conditions. Then, carbon (acetate or propionate) and phosphorus sources were added to the COD concentration of 100 mg/L and the PO_4_^3−^-P concentration of 10 mg/L for 1 h of anaerobic condition. Thereafter, nitrogen gas was stopped and the air was pumped to obtain the aerobic condition for 2 h. During the test, pH was manually maintained at 7.0 ± 0.1 by the addition of NaOH or HCl. Temperature were maintained at 20 °C. Samples were periodically collected throughout the test and filtered through 0.45 μm filter membranes to determine COD and PO_4_^3−^-P concentration. Sludge samples collected at the beginning and end of the test were collected to measure sludge concentration (i.e., mixed liquid suspended solids (MLSS) and mixed liquids volatile suspended solids (MLVSS)). The specific kinetic rate, such as anaerobic P release rate, substrate uptake rate, and aerobic P uptake rate, is expressed as the slope of the linear regression equation for the concentration–time plot (i.e., volumetric rate), dividing by MLVSS concentration. The P release–substrate uptake ratio is calculated as the mass of phosphorus released divided by the mass of substrate removed from the bulk solution.

### 2.3. Microbial Community Analysis

During the experiment, activated sludge samples were collected from the reactor at each phase for DNA extraction and 16S rRNA gene amplicon sequencing. Genomic DNA was extracted from each sample using the DNeasy PowerSoil Kit (QIAGEN, Inc., Hilden, Germany). Primers 338F (5′-ACTCCTACGGGAGGCAGCA-3′) and 806R (5′-GGACTACHVGGGTWTCTAAT-3′) were used for PCR amplification of the bacterial 16S rRNA gene. After the amplification, products were purified using Agencourt AMPure beads (Beckman Coulter, Indianapolis, IN, USA) and quantified using a PicoGreen dsDNA Assay Kit (Invitrogen, Carlsbad, CA, USA). Sequencing libraries were created and the Illumina MiSeq platform was used for 16S rRNA gene amplicon sequencing (Shanghai Personal Biotechnology Co., Ltd., Shanghai, China). Finally, bioinformatics analysis of the sequencing data was performed using QIIME (v1.8.0) software. The analysis of the relative abundance of the nutrient removal-related functional microorganisms was conducted by analyzing the sequencing data with the assistance of the Activated Sludge Microbial Database (MiDAS) [31] linking the taxonomy with multiple metabolic functions (e.g., nutrient removal, fermentation, etc.) [32].

### 2.4. Chemical Analyses

COD was determined by the potassium dichromate method. NH_4_^+^-N was determined using the Nessler reagent (Hach, Loveland, CO, USA). NO_3_^−^-N and NO_2_^−^-N were determined using ultraviolet spectrophotometry. PO_4_^3−^-P was determined using ammonium molybdate reagent (Hach, Loveland, CO, USA). TN and TP were determined by the alkaline persulfate digestion method. Removal efficiency (%) is calculated as the difference between the pollutant (COD, NH_4_^+^-N, TN, and PO_4_^3−^-P) concentration in the influent and effluent, divided by the pollutant concentration in the influent. The acetate or propionate concentration in the single carbon-feeding batch tests was determined via measuring COD in the supernatant and calculated based on the theoretical COD equivalents (i.e., 1.07 mg COD/g acetate and 1.52 mg COD/g propionate). MLSS and MLVSS were determined according to Standard Methods [33]. The modified thermal extraction method described by Domínguez et al. [34] was used to extract soluble microbial products (SMP) and extracellular polymeric substances (EPS) from the mixed liquor. The concentrations of proteins, polysaccharides, lipids, humic acids, and DNA in SMP; loosely bound EPS (LB-EPS); and tightly bound EPS (TB-EPS) were measured. A modified Lowry method [35,36] was employed to quantify proteins and humic acids. The anthrone method [37] was used to analyse polysaccharide concentration. The lipid concentration was determined using the sulfo-phospho-vanillin method [38], and DNA concentration was determined using the diphenylamine colorimetric method [39]. All samples were analysed in triplicate. 

## 3. Results

### 3.1. Pollutant Removal Performance

#### 3.1.1. COD Removal Performance

**Figure 1:**
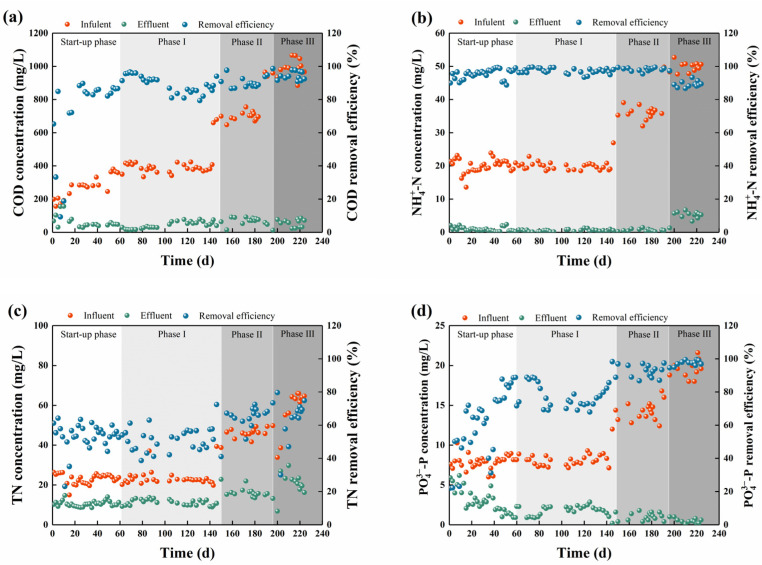
The removal performance of (**a**) COD, (**b**) NH_4_^+^-N, (**c**) TN, and (**d**) PO_4_^3−^-P during the experiment.

The variations in the COD removal performance in each phase during the experiment are shown in Figure 1a. In the initial start-up phase (0–16 days), fluctuations in the COD removal performance were observed. The effluent COD (COD_eff_) concentration was 97 ± 51 mg/L with an average removal efficiency of 47.3%. On days 17 and 41, the COD_inf_ concentration increased from 193 ± 30 mg/L to 289 ± 20 and 333 ± 55 mg/L, respectively, and the removal performance gradually improved, which is possibly related to the consequently increased microbial growth and substrate degradation rates [40]. In Phase I, II, and III when the COD_inf_ concentrations increased to 388 ± 25, 696 ± 27, and 983 ± 49 mg/L, respectively, the average removal efficiencies still reached 89.0%, 89.8%, and 94.5%, respectively. As the easily biodegradable matter, the VFAs (i.e., acetate and propionate) in the influent are expected to be fast degraded during the SBR cycle. While for the complex carbon source that is mainly composed of proteins and amino acids (i.e., casein acid hydrolysate) and commonly exists in dairy wastewater [41], its biodegradation would be slower and relies on the specific microorganisms capable of utilising amino acids [42]. Our results showed that the A/O-SBR was effective in degrading different organic matter under high influent loading conditions. This is possibly related to the increase in active biomass, as the sludge concentration increased from ~5 to ~7 g/L during the experiment.

#### 3.1.2. Nitrogen Removal Performance

**Figure 2:**
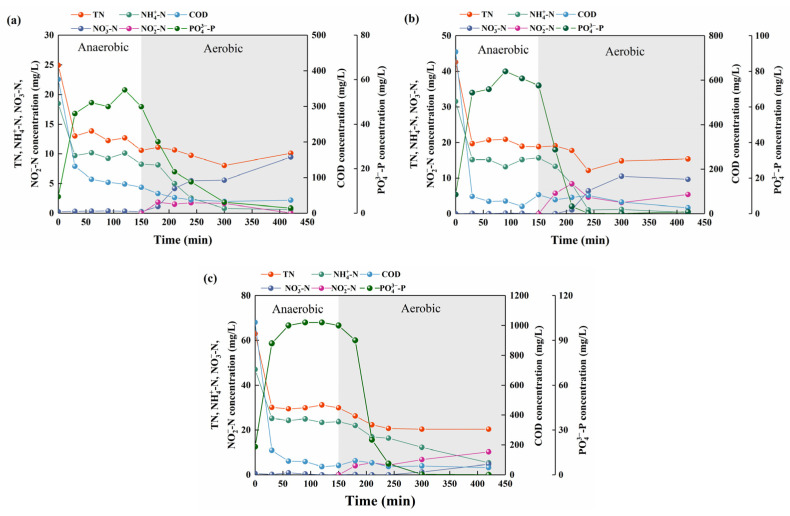
Profiles of COD, TN, NH_4_^+^-N, NO_3_^−^-N, NO_2_^−^-N, and PO_4_^3−^-P concentrations in a typical cycle of SBR: (**a**) Phase I, (**b**) Phase II, and (**c**) Phase III.

During the start-up phase, the average NH_4_^+^-N removal efficiency of the SBR was 93.1%, indicating that the reactor achieved good nitrogen removal performance (Figure 1b). NH_4_^+^-N removal was also consistently good in Phase I and II, with removal efficiencies of 97.2% and 98.2%, respectively. As the NH_4_^+^-N_inf_ concentration increased to 49.9 ± 1.3 mg/L in Phase III, the effluent NH_4_^+^-N (NH_4_^+^-N_eff_) concentration increased obviously to 5.1 ± 1.8 mg/L, with a declined average removal efficiency of 89.8%. This may be related to the excessive organic matters in influent that were not completely degraded during the anaerobic phase (Figure 2). The residual organic matter could promote the proliferation of other heterotrophic organisms (OHOs) during the aerobic phase, leading to intense competition between nitrifying and heterotrophic bacteria for oxygen and space [43]. The organic loading condition would also lead to inhibited nitrification due to the inactivation of enzymes in the nitrification process [44]. Meanwhile, the increased NH_4_^+^-N load in Phase III may exceed the removal capacity of nitrifying bacteria in the reactor, thus leading to an increase in the NH_4_^+^-N_eff_ concentration. The denitrification performance of the SBR gradually increased under the three different loading conditions (Figure 1c), with the average removal efficiencies of 51.8%, 63.5%, and 64.5%, respectively, which should be attributed to the increased organic loads providing sufficient electron donors to denitrifiers [45].

#### 3.1.3. Phosphorus Removal Performance

The biological phosphorus removal performance in SBR during the experiment is shown in Figure 1d. The average PO_4_^3−^-P removal efficiency at the early stage of the start-up phase (days 1–16) was only 42.0%, and the effluent PO_4_^3−^-P (PO_4_^3−^-P_eff_) concentration was 4.7 ± 1.3 mg/L with large fluctuations. This may be related to the relatively long sludge age (~25 d). In the middle stage of the start-up phase (days 17–40), the SRT of the reactor was reduced to ~14 d by increasing the amount of waste sludge, which lead to ascending phosphorus removal performance. During the subsequent phases, as the influent loads elevate, the average PO_4_^3−^-P removal efficiencies steadily increased to 77.4%, 92.3%, and 97.5% in Phase I, II, and III, respectively. The highest and most stable PO_4_^3−^-P removal performance occurred in Phase III, with an average PO_4_^3−^-P_eff_ concentration of 0.56 mg/L (Figure S2), which is superior to another study treating dairy manure wastewater (PO_4_^3−^-P_inf_: 51.1 ± 23.0 mg/L; PO_4_^3−^-P removal efficiency: 59%) [46]. Yuan et al. [18] also obtained similar effective performance in a lab-scale SBR treating synthetic wastewater (PO_4_^3−^-P_inf_: 40.0 mg/L; PO_4_^3−^-P removal efficiency: 99.5 ± 0.8%), yet only used simple organic matter (i.e., acetate and propionate) as carbon sources. The effective PO_4_^3−^-P removal in this study should be attributed to the high influent organic load, which reduced the competition for carbon sources between PAOs and other heterotrophic bacteria (e.g., denitrifying bacteria and OHOs), making more available carbon sources to PAOs. 

In addition, the amounts of proteins, polysaccharides, and humic acids in the EPS of the activated sludge also largely increased with the increase in the COD_inf_ load (Figure S3). Recent studies have demonstrated that EPS plays an important role in P removal by EBPR sludge, mainly due to its large specific surface area and abundant functional groups (e.g., hydroxyl, carboxyl, sulfonate, etc.) capable of adsorbing phosphorus [47,48]. Meanwhile, EPS was considered to have a positive effect on sludge flocculation, promoting cell aggregation during the sludge granulation process [49]. Therefore, the contribution of EPS to the EBPR process treating high-strength wastewater needs further investigation. 

Considering the removal performance for each pollutant, the A/O-SBR used in this study could effectively treat different levels of pre-fermented high-strength wastewater. Although the NH_4_^+^-N_eff_ concentration increased in Phase III, it should be possible to achieve improved nitrification performance via extending HRT and reducing residual organic matter in the aerobic phase. Further optimisation studies of the A/O-SBR are warranted to enhance its pollutant removal performance and expand its application range in treating wastewaters with varying influent loads.

### 3.2. Microbial Activities

#### 3.2.1. Nitrogen Removal Activity

The effects of different influent loads on the pollutant treatment process during a typical cycle of SBR (anaerobic: 2.5 h; aerobic: 4.5 h) were analysed in different phases (Figure 2). The TN concentration decreased gradually throughout the typical cycle, and the effluent TN (TN_eff_) concentration decreased with an increase in the concentrations of COD_inf_ and influent TN (TN_inf_), indicating that the sludge had efficient denitrification capacity under high-strength influent conditions. The NH_4_^+^-N concentration decreased during the aerobic period of the typical cycle, and the NO_3_^−^-N concentration increased accordingly. Along with the gradually elevated NH_4_^+^-N supply in the influent, the average specific ammonia oxidation rate (AOR) increased from 0.51 mg N/(g VSS·h) in Phase I to 0.72 mg N/(g VSS·h) in Phase III (Table S1). Notably in Phase III, a higher NH_4_^+^-N concentration (5.4 mg/L) was detected at the end of the aerobic period. This incomplete oxidation of NH_4_^+^-N should be related to the high NH_4_^+^-N_inf_ load and the relatively low ammonia-oxidising bacteria (AOB) activity [50]. Regarding the specific nitrite oxidation rate (NOR), the average value decreased largely from 0.61 mg N/(g VSS·h) in Phase I to 0.18 mg N/(g VSS·h) in Phase III (Table S1), which is much lower than the AOR in Phase III (0.72 mg N/(g VSS·h)). Both AOR and NOR detected in this study are much lower than the values exhibited in other activated sludge systems (Table S3), which is probably due to the pretty low AOB and NOB abundance (See Section 4.1).

Meanwhile, substantially increased NO_2_^−^-N concentrations were observed at the end of the aerobic period in Phase II (5.4 mg/L) and III (10.3 mg/L) (Figure 2), with the corresponding NO_2_^−^-N accumulation rates (NAR) of 36% and 68%. This indicates that the high-strength influent conditions had a more pronounced inhibition effect on nitrite-oxidising bacteria (NOB) than on AOB, resulting in the NO_2_^−^-N accumulation in the effluent. 

#### 3.2.2. Phosphorus Removal Activity

EBPR characteristics were observed during the typical cycle of SBR (Figure 2). The COD concentration in the SBR decreased sharply from 376.0–1020.8 mg/L to 55.4–95.1 mg/L within the first 60 min of the anaerobic period, indicating that most of the organic matter in the influent could be rapidly degraded under the anaerobic conditions. Correspondingly, the PO_4_^3−^-P concentration largely increased to 49.7–100.0 mg/L and decreased obviously to 0.1–2.3 mg/L in the subsequent aerobic period. The anaerobic P release amount (PRA) were 40.4, 61.2, and 81.2 mg/L and the aerobic P uptake amount (PUA) values were 45.6, 71.0, and 99.9 mg/L for Phase I, II, and III, respectively, indicating that the increase in organic load promoted the P release and uptake capacities of the activated sludge in all phases.

To further investigate the effects of different carbon sources on EBPR activity, the batch tests fed with acetate or propionate were conducted using sludge samples from different phases (Figures S4 and S5). All the specific kinetic rates and stoichiometric ratios were within the range observed in other EBPR systems treating conventional low-strength municipal wastewater (Table 2). The anaerobic P release to acetate uptake (P/HAc) ratio, which is an indicator of PAO activity and abundance [51], decreased from 0.64 P-mol/C-mol in Phase I to 0.38 P-mol/C-mol in Phase III (Table 2). This indicates the presence of competition between PAOs and GAOs for carbon sources, potentially leading to decreased abundance of acetate-utilising PAOs (e.g., *Accumulibacter*) (as shown in Section 4.3). In contrast, the anaerobic P release to propionate uptake (P/HPr) ratio increased from 0.73 P-mol/C-mol in Phase I to 0.81 P-mol/C-mol in Phase III, and the P/HPr ratio in each phase was higher than the P/HAc ratio.

### 3.3. Microbial Community Structure

#### 3.3.1. Microbial Diversity

The 16S rRNA gene amplicon sequencing data were analysed to obtain operational taxonomic units (OTUs) based on clustering at a similarity level of 0.97. Alpha diversity indices, including the Chao1, ACE, Shannon, and Gini–Simpson indices, were calculated for each activated sludge sample based on the OTUs (Table 3) [57]. The Good’s coverage index was higher than 0.99 for all 4 samples, indicating the current sequences represented the majority of the bacterial community. The sludge sample in Phase III had the highest diversity index, which is probably due to the increased influent load providing sufficient nutrients for the growth of microorganisms, as well as mitigating the competition between different microorganisms to a certain extent.

#### 3.3.2. Microbial Community Composition

**Figure 3:**
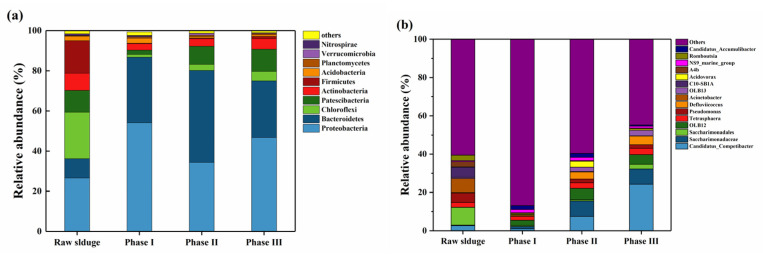
Relative abundances of microbial community composition at the (**a**) phylum and (**b**) genus levels during the experiment.

The relative abundance of microorganisms at the phylum levels in different phases during the experiment is shown in Figure 3a. In the raw sludge, Proteobacteria and Bacteroidetes were the dominant groups, with the relative abundance of 26.6% and 9.6%, respectively. After running under different loading conditions, the relative abundance of Proteobacteria and Bacteroidetes increased significantly, reaching 34.4%–54.1% and 28.1%–45.8%, respectively. Proteobacteria are dominant in many activated sludge systems, and include many microorganisms associated with organic matter degradation and nutrient cycling (e.g., some known denitrifying bacteria and PAOs) [58,59]. Lawson and Strachan [60] found that certain bacteria in Bacteroidetes also play an important role in denitrification. At the genus level, *Thauera*, *Terrimonas*, and *Haliangium* were the dominant genera in Phase I, while *Saccharimonadaceae*, *Defluviicoccus*, *Flavobacterium*, *Competibacter*, and *Tetrasphaera* were dominated in Phase II and III when the influent load reached higher levels (Figure 3b). This indicates that the microbial community structure changed largely and continuously from the raw sludge after reactor operation, which is probably related to the elevated organic and nutrient loads providing selection pressures to the community.

## 4. Discussion

### 4.1. Impact of Organic Load on Nitrogen-Removal-Related Microorganisms

**Figure 4:**
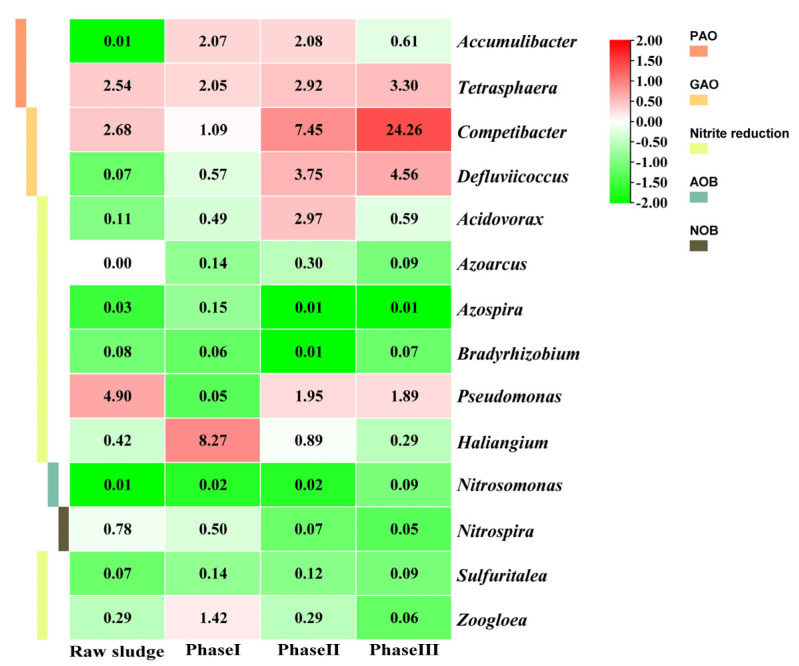
Relative abundance of known functionally relevant microorganisms for nitrogen and phosphorus removal during the experiment.

The changes in functional microorganisms during the experiment (Figure 4) revealed that the relative abundance of AOB (i.e., *Nitrosomonas*) increased from 0.02% in Phase I to 0.09% in Phase III, whereas the relative abundance of NOB (i.e., *Nitrospira*) decreased substantially from 0.50% in Phase I to 0.05% in Phase III, which is consistent with the results obtained in the batch test (see Section 3.2.1). This distinct shift of the nitrifying bacterial population was possibly related to the increased influent load. For the typical WWTPs treating municipal wastewater, the COD level in the aerobic zone is often low since the influent organic matters were mainly degraded in the ahead anaerobic/anoxic zone. Therefore, less attention has been paid to the effect of organic loads on nitrifying bacteria. However, the organic matter concentration is significantly higher in high-strength wastewater, and their residues in the aerobic zone may impact nitrifying bacteria [44]. It has been found that the addition of small-molecule VFAs (i.e., formic, acetic, propionic, or butyric acid) in the aerobic phase inhibits NOB activity without affecting AOB activity, whereas the addition of valeric or capric acid negatively affects both AOB and NOB activities [61]. In this study, the gradual increase in acetate/propionate-dominated organic load might lead to higher suppression and out-selection of NOB populations compared to AOB. 

The novel shortcut nitrification-denitrification (SND) and partial nitrification/anammox (PN/A) processes in the mainstream system have been receiving much attention due to the great savings in oxygen/energy consumption and carbon source utilisation [62,63]. However, studies on these processes have mainly focused on their application in wastewater treatment with low concentrations of carbon sources and/or low COD/N ratios, and very limited research has been conducted on the potential application of shortcut nitrification-based processes in high-strength wastewater treatment [64,65,66]. The simultaneous accumulation of NO_2_^−^-N and NH_4_^+^-N in the A/O-SBR treating high-strength wastewater (Figure 2c), as observed in this study, provides a preliminary basis for the development of a mainstream SND- or PN/A-based process, which requires further in-depth and systematic studies. 

### 4.2. Impact of Carbon Sources on Phosphorus Removal Activity

Previous studies have shown that acetate (49%–71% of total VFA) and propionate (24%–33% of total VFA) are the two most representative VFAs in domestic wastewater [67]. The propionate uptake rate of typical GAOs (i.e., *Competibacter*), which compete with typical PAOs (i.e., *Accumulibacter*) for carbon sources, was much lower than that of acetate [68], which explains the better EBPR activity in the reactor when propionate was used as the carbon source [69,70,71]. The differential PAO activities observed in the batch tests with different carbon sources (Table 2) suggest that the sludge may contain a large number of *Competibacter* GAOs that primarily utilise acetate (as shown in Figure 4), which to some extent affects the uptake of acetate by *Accumulibacter* PAOs and thus reduces the corresponding EBPR activity. However, compared to those in other studies [29,72,73], the P/HAc and P/HPr ratios in this study were consistently high, indicating that the presence of GAOs did not have a significant negative effect on the EBPR activity. In addition, the complex substrate (casein acid hydrolysate) in the influent was also available to other PAOs (e.g., *Tetrasphaera*) [74,75] and thus contributed to the overall EBPR activity.

### 4.3. Impact of Organic Load on Phosphorus-Removal-Related Microorganisms

The treatment performance of activated sludge systems depends heavily on the coordination among different functionally relevant microorganisms, and the microbial community composition and diversity are closely related to the system stability [76,77]. For EBPR-related functional microorganisms, the relative abundance of *Competibacter* GAOs with a higher acetate uptake rate increased significantly from 1.1% in Phase I to 24.3% in Phase III, whereas the relative abundance of *Defluviicoccus* GAOs with higher propionate uptake rate increased from 0.6% to 4.6%. Under low substrate concentration conditions, the presence of GAOs inhibited the phosphorus uptake activity of PAOs due to their competition for VFAs [78], while the GAO abundance would increase with the elevated substrate concentration in the influent [51,52]. Therefore, most EBPR studies have focused on how to limit the proliferation of GAOs through various pathways. The suitable COD/P ratio for EBPR activity was found to be 15:1–25:1 in low-strength wastewater [23,51], while higher COD/P ratios (e.g., 50:1) will be detrimental to the growth of PAOs but beneficial to GAOs [12]. However in this study, when the influent organic load was elevated to a sufficiently high level with a COD/P ratio of 50:1, EBPR activity was not affected (see Section 3.2.2) despite a significant increase in the GAO abundance. It suggests that there may not be significant substrate competition between PAOs and GAOs in the systems treating high-strength wastewater with various types of carbon sources [52,79]. Notably, the relative abundance of *Accumulibacter* PAOs decreased from 2.1% in Phase I to 0.6% in Phase III, whereas the relative abundance of *Tetrasphaera* PAOs increased from 2.0% to 3.3%. *Tetrasphaera* is a PAO that can utilise complex carbon sources (e.g., protein), whereas *Competibacter* is generally considered to utilise simple carbon sources only [79]. The different carbon source utilisation capabilities of the two groups would be beneficial in mitigating the PAO–GAO competition, and therefore high EBPR activity and phosphorus removal performance could be achieved in Phase III. 

Another study proved that some species of *Competibacter* are denitrifying GAOs (DGAOs) [80]. The VFAs produced by *Tetrasphaera* when fermenting complex carbon sources would potentially provide additional substrate for *Competibacter*. The consequently enriched *Competibacter* DGAOs would promote nitrogen removal performance. Therefore, the coexistence of *Tetrasphaera* PAOs and *Competibacter* DGAOs, as observed in this study, may not only avoid competition for carbon sources but also synergistically promote nitrogen and phosphorus removal, as observed in a previous study [81]. Further investigation is warranted to determine how this synergy can be applied in a continuous flow reactor treating high-strength wastewater and coupled with the shortcut nitrification-based process discussed in Section 4.1.

## 5. Conclusions

(1) The anaerobic/aerobic SBR could effectively treat pre-fermented high-strength wastewater at different levels. The removal efficiencies of COD, TN, and PO_4_^3−^-P were 94.5%, 64.5%, and 97.5%, respectively.

(2) The NOB activity and population were severely suppressed under high-strength influent loading conditions, achieving partial nitrification with simultaneous accumulation of NH_4_^+^-N and NO_2_^−^-N in the effluent. Increased organic load promoted the anaerobic PRA and aerobic PUA. EBPR activity was higher when propionate was used as the carbon source.

(3) Sufficient organic load in the high-strength wastewater obviously mitigated the competition for substrate among PAOs and GAOs. The coexistence of *Tetrasphaera* and *Competibacter* DGAOs observed in the system would enable a synergistic effect on the simultaneous nitrogen and phosphorus removal.

## Figures and Tables

**Table 1:** Main components of synthetic pre-fermented high-strength wastewater.

Influent Concentration	Start-Up Phase			Phase I Days 61–142	Phase II Days 143–183	Phase III Days 184–224
	Days 1–16	Days 17–40	Days 41–60			
COD (mg/L)	192 ± 27	289 ± 20	333 ± 55	388 ± 25	696 ± 27	983 ± 49
TN (mg/L)	23.1 ± 4.3	22.1 ± 2.0	24.1± 1.0	23.3 ± 3.1	43.5 ± 7.3	56.1 ± 10.4
NH_4_^+^-N (mg/L)	21.4 ± 0.7	19.8 ± 2.3	19.3 ± 0.9	19.8 ± 1.1	35.4 ± 2.9	49.9 ± 1.3
PO_4_^3−^-P (mg/L)	8.2 ± 1.5	7.6 ± 2.3	8.3 ± 0.4	8.1 ± 0.6	13.9 ± 1.0	19.2 ± 1.5
COD/N ratio	9.0	14.6	17.2	19.6	19.7	19.7
COD/P ratio	23.4	38.0	40.1	47.9	50.1	51.2

**Table 2:** Specific kinetic rates and stoichiometric ratios observed in the P release and uptake batch tests fed with different carbon sources during the experiment.

Carbon Source		P Release Rate [mg P/(g VSS·h)]	Substrate Uptake Rate [mg C/(g VSS·h)]	P Uptake Rate [mg P/(g VSS·h)]	P Release/Substrate Uptake Ratio (P-mol/C-mol)	Reference
Acetate	Phase I	10.3	6.2	4.1	0.64	This study
	Phase II	4.7	6.4	1.2	0.30	This study
	Phase III	7.0	7.3	3.2	0.38	This study
	Full-scale sludge	5.6-31.9	16.1-42.5	2.4-9.7	0.29-0.75	[52]
	Full-scale sludge	2.8-5.3	7.7-24.9	0.6-2.6	0.16-0.54	[53]
	Lab-scale sludge	4.4-50.6	7.7-32.7	9.8-23.8	0.22-0.60	[54]
Propionate	Phase I	9.8	5.2	3.7	0.73	This study
	Phase II	7.3	4.7	1.5	0.60	This study
	Phase III	6.4	3.0	2.6	0.81	This study
	Lab-scale sludge	13.6	36.7	18.6	0.27	[55]
	Full-scale sludge	-	-	-	0.38-0.60	[56]

**Table 3:** Alpha diversity indices in activated sludge samples during the experiment.

Samples	Observed Species	Good’s Coverage	Pielou’s Evenness	Chao1	Gini–Simpson	Shannon
Raw sludge	2768	0.990	0.792	3024	0.994	9.063
Phase I	3035	0.995	0.736	3090	0.984	8.515
Phase II	3070	0.996	0.698	3085	0.968	8.090
Phase III	3065	0.990	0.723	3208	0.986	8.378

## Data Availability

Not applicable.

## Supplementary Materials

The following supporting information can be downloaded at: https://www.mdpi.com/article/10.3390/ijerph19095653/s1, Figure S1: Schematic diagram of the SBR system; Figure S2: Cumulative frequency curves of (a) COD, (b) NH_4_^+^-N, (c) TN and (d) PO_4_^3−^-P concentrations in the effluent during the experiment; Figure S3: The concentrations of (a) proteins, (b) humic acids, and (c) polysaccharides in the soluble microbial products (SMP), loosely bound (LB-EPS) and tightly bound extracellular polymeric substances (TB-EPS) of the activated sludge during the experiment; Figure S4: Profiles of PO_4_^3−^-P and COD during P release and uptake batch tests fed with acetate in (a) Phase I, (b) Phase II, and (c) Phase III; Figure S5: Profiles of PO_4_^3−^-P and COD during P release and uptake batch tests fed with propionate in (a) Phase I, (b) Phase II, and (c) Phase III. Table S1: Summary of COD/P and COD/N ratios in high-strength dairy and manure wastewater [18,19,26,28,46,82]; Table S2: Component and concentrations of other trace elements in the synthetic pre-fermented high-strength wastewater; Table S3: Component and concentrations of other trace elements in the synthetic pre-fermented high-strength wastewater [83,84]; Table S4: Average specific ammonia oxidation rate (AOR) and specific nitrite oxidation rate (NOR) in typical cycles of SBR reactor during the experiment.
