# Supplementary material for: Partial Nitrification and Enhanced Biological Phosphorus Removal in a Sequencing Batch Reactor Treating High-Strength Wastewater
Source: Int J Environ Res Public Health. 2022 May 6;19(9):5653. doi: 10.3390/ijerph19095653 (PMC9105176; doi:10.3390/ijerph19095653)
Supplement: Supplementary file 1 [file ijerph-19-05653-s001.zip › ijerph-1680529-supplementary.pdf]

Supplementary Information for:

Partial Nitrification and Enhanced Biological Phosphorus Removal in a Sequencing Batch Reactor Treating High-Strength Wastewater

Xiaojun Feng [1], Yishi Qian [1,2], Peng Xi [1], Rui Cao [3], Lu Qin [3], Shengwei Zhang [3], Guodong Chai [3], Mengbo Huang [3], Kailong Li [3], Yi Xiao[3], Lin Xie[3], Yuxin Song[3] and Dongqi Wang[3,4,5,*]

[1] Xi'an Modern Chemistry Research Institute, Xi'an 710065, China

[2] School of Energy and Power Engineering, Xi'an Jiaotong University, Xi'an 710049, China

[3] Department of Municipal and Environmental Engineering, Xi'an University of Technology, Xi'an, Shaanxi 710048, China

[4] State Key Laboratory of Eco-hydraulics in Northwest Arid Region, Xi'an University of Technology, Xi'an, Shaanxi 710048, China

[5] Shaanxi Key Laboratory of Water Resources and Environment, Xi'an University of Technology, Xi'an, Shaanxi 710048, China

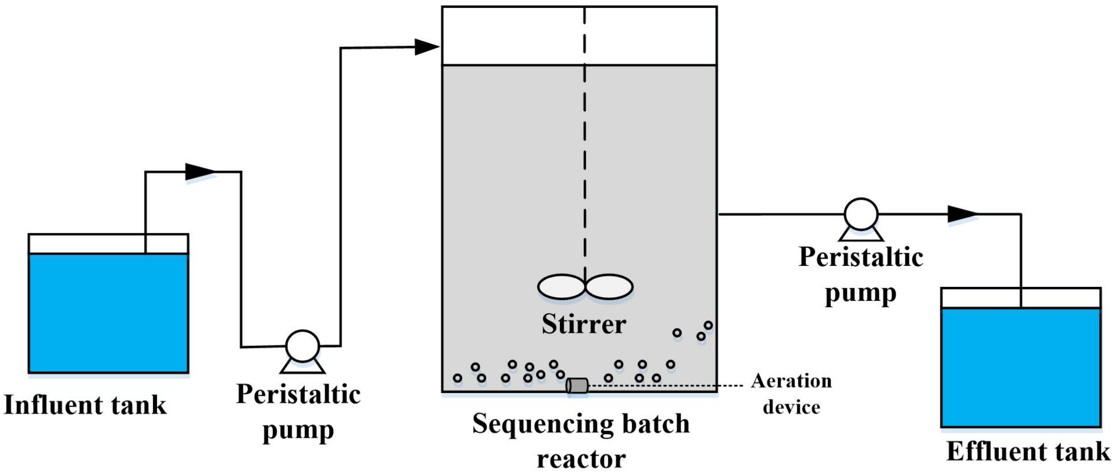

Figure S1. Schematic diagram of the SBR system.

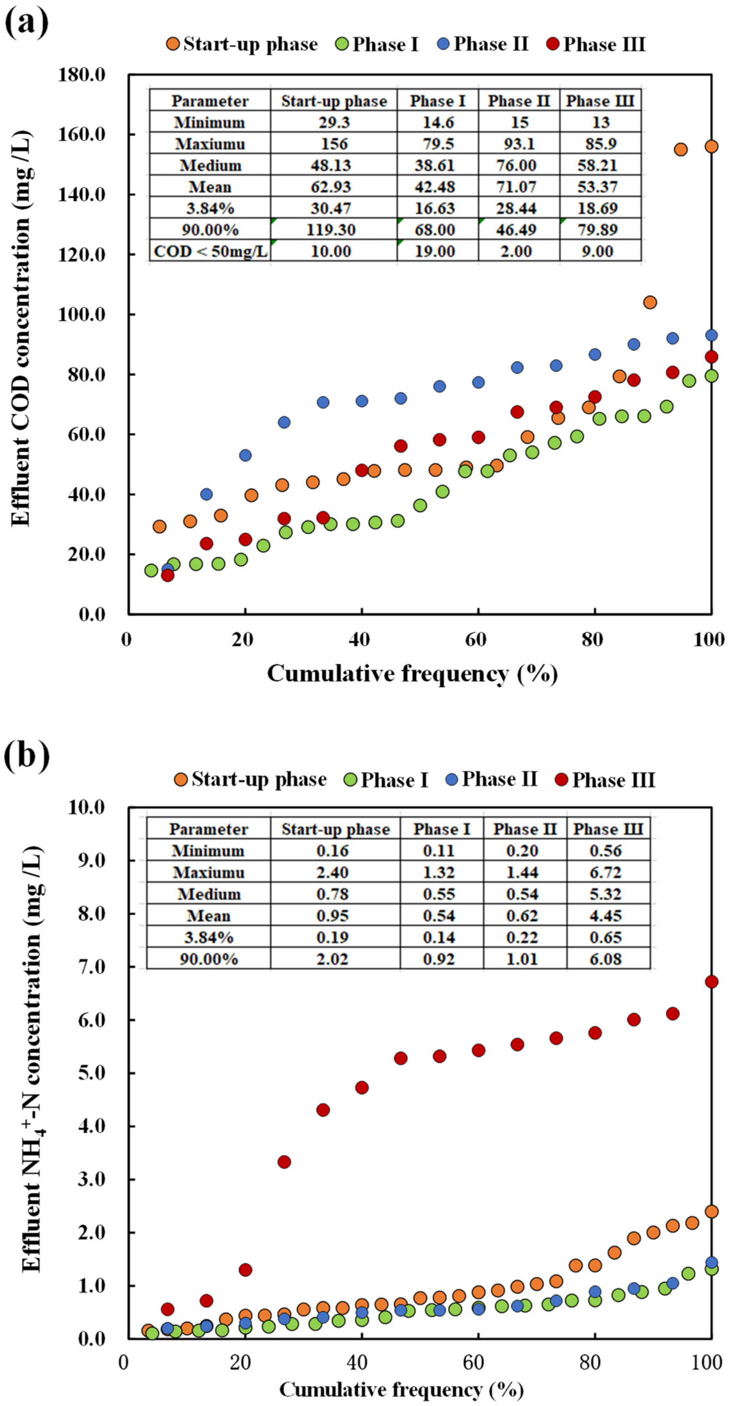

Parameter	Start-up phase	Phase I	Phase II	Phase III
Minimum	29.3	14.6	15	13
Maxiumu	156	79.5	93.1	85.9
Medium	48.13	38.61	76.00	58.21
Mean	62.93	42.48	71.07	53.37
3.84%	30.47	16.63	28.44	18.69
90.00%	119.30	68.00	46.49	79.89
COD < 50mg/L	10.00	19.00	2.00	9.00

Parameter	Start-up phase	Phase I	Phase II	Phase III
Minimum	0.16	0.11	0.20	0.56
Maxiumu	2.40	1.32	1.44	6.72
Medium	0.78	0.55	0.54	5.32
Mean	0.95	0.54	0.62	4.45
3.84%	0.19	0.14	0.22	0.65
90.00%	2.02	0.92	1.01	6.08

(c)

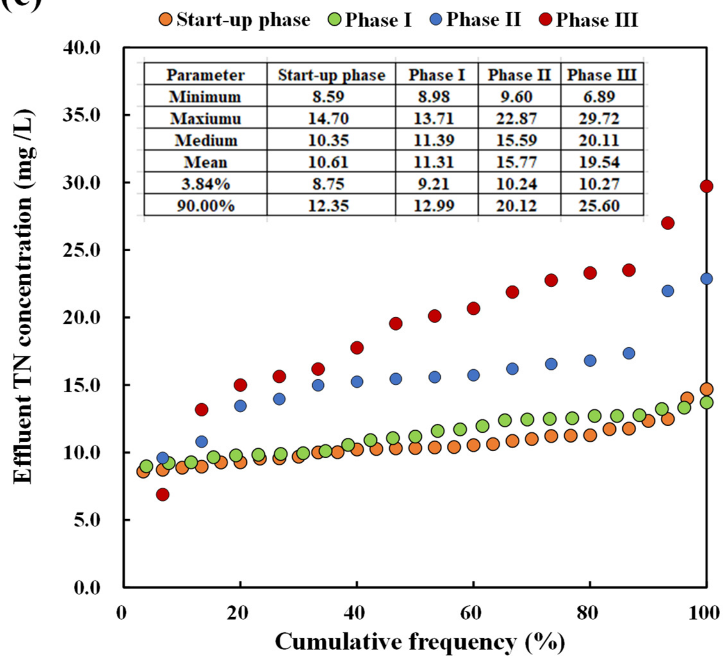

Parameter	Start-up phase	Phase I	Phase II	Phase III
Minimum	8.59	8.98	9.60	6.89
Maxiumu	14.70	13.71	22.87	29.72
Medium	10.35	11.39	15.59	20.11
Mean	10.61	11.31	15.77	19.54
3.84%	8.75	9.21	10.24	10.27
90.00%	12.35	12.99	20.12	25.60

(d)

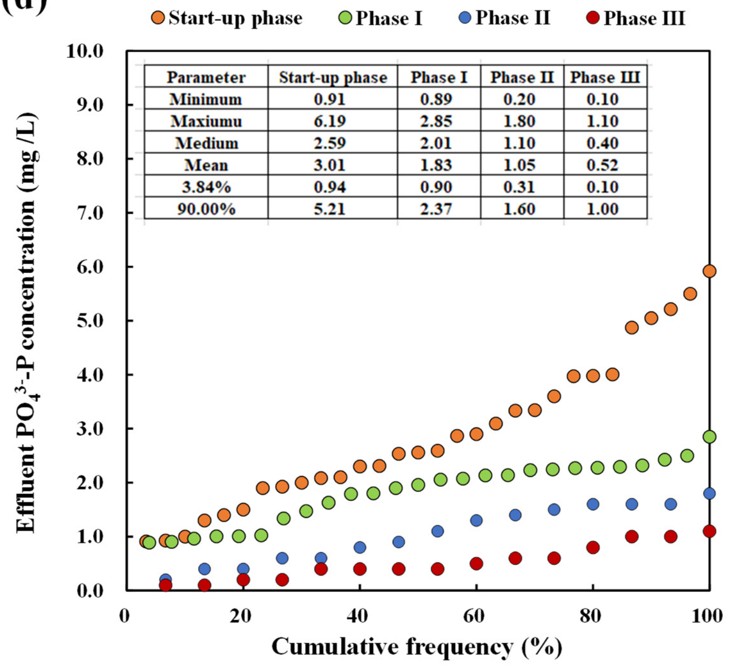

Parameter	Start-up phase	Phase I	Phase II	Phase III
Minimum	0.91	0.89	0.20	0.10
Maxiumu	6.19	2.85	1.80	1.10
Medium	2.59	2.01	1.10	0.40
Mean	3.01	1.83	1.05	0.52
3.84%	0.94	0.90	0.31	0.10
90.00%	5.21	2.37	1.60	1.00

Figure S2. Cumulative frequency curves of (a) COD, (b) NH_4^+-N, (c) TN and (d) PO_4^{3-}-P concentrations in the effluent during the experiment.

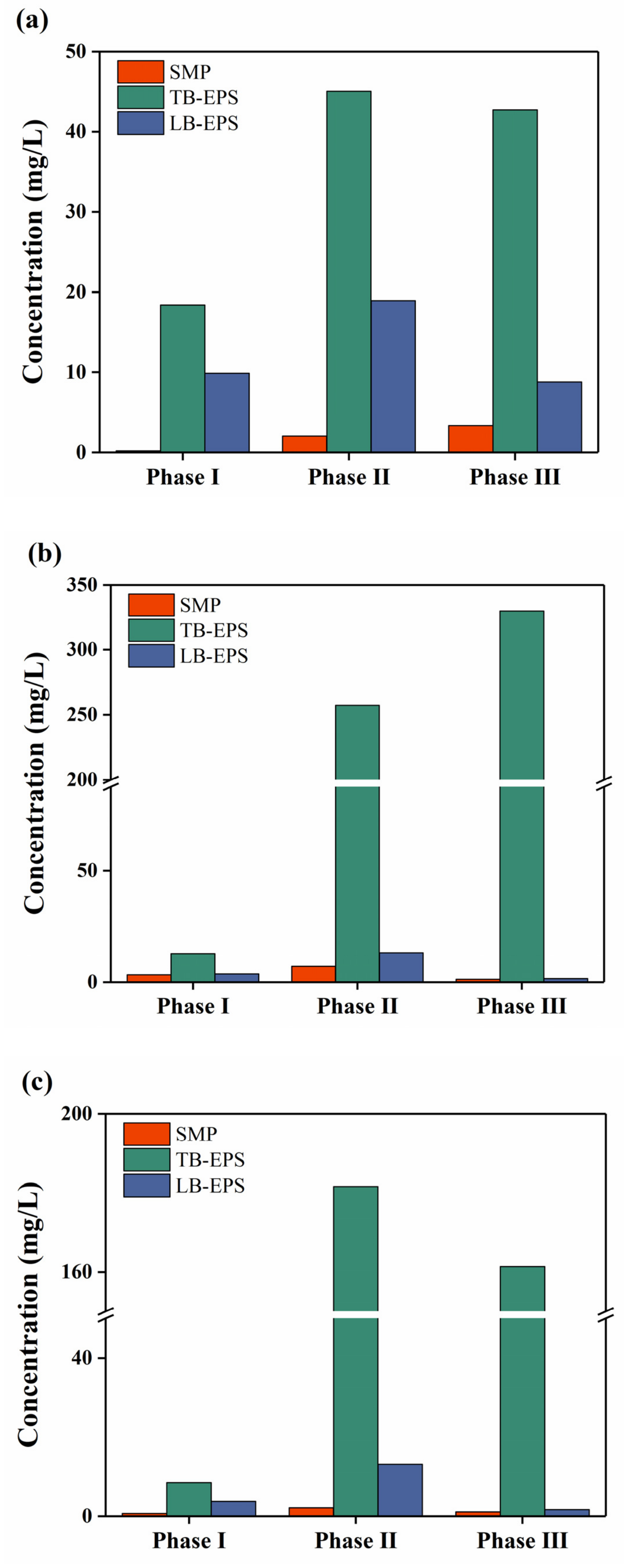
(a)
Concentration (mg/L)
0
10
20
30
40
50
SMP
TB-EPS
LB-EPS
Phase I
Phase II
Phase III
(b)
Concentration (mg/L)
0
50
200
250
300
350
SMP
TB-EPS
LB-EPS
Phase I
Phase II
Phase III
(c)
Concentration (mg/L)
0
40
160
200
SMP
TB-EPS
LB-EPS
Phase I
Phase II
Phase III

Figure S3. The concentrations of (a) proteins, (b) humic acids, and (c) polysaccharides in the soluble microbial products (SMP), loosely bound (LB-EPS) and tightly bound extracellular polymeric substances (TB-EPS) of the activated sludge during the experiment.

(a)

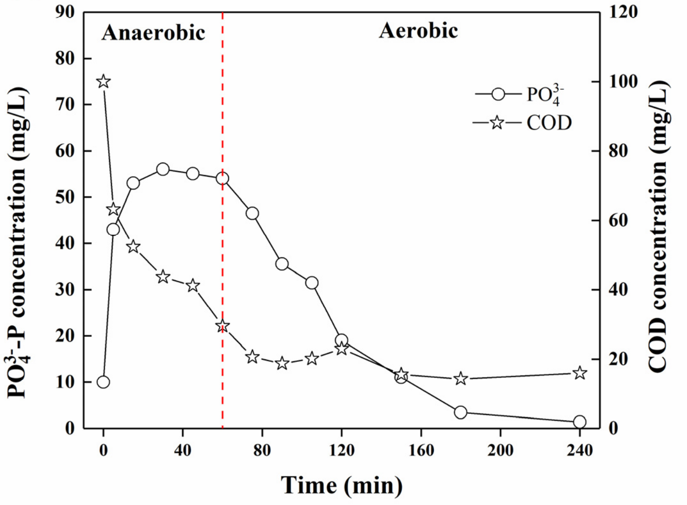

(b)

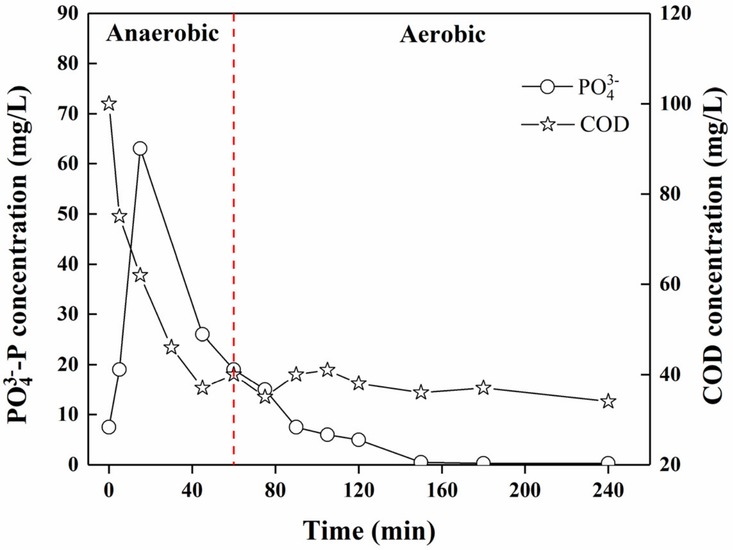

(c)

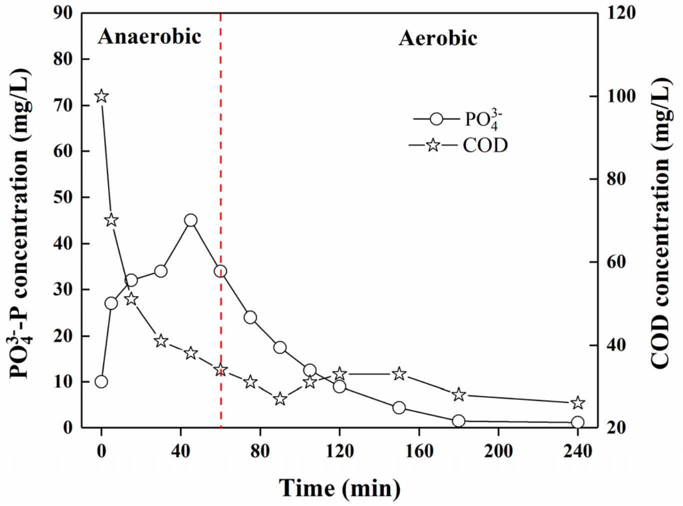

Figure S4. Profiles of PO_4^{3-}-P and COD during P release and uptake batch tests fed with acetate in (a) Phase I, (b) Phase II, and (c) Phase III.

(a)

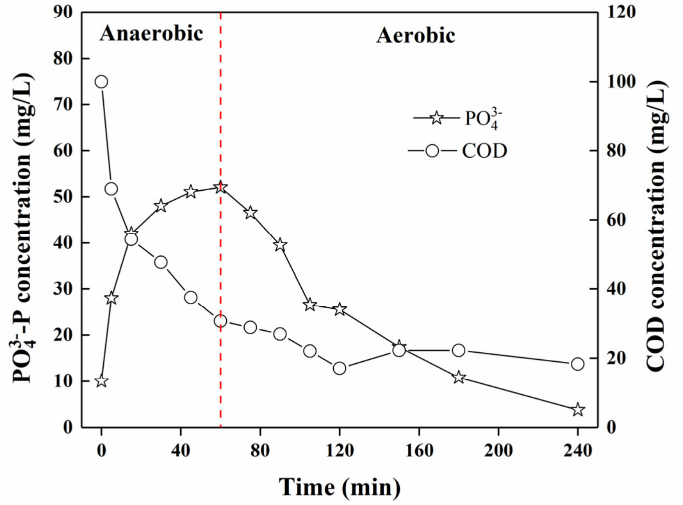

(b)

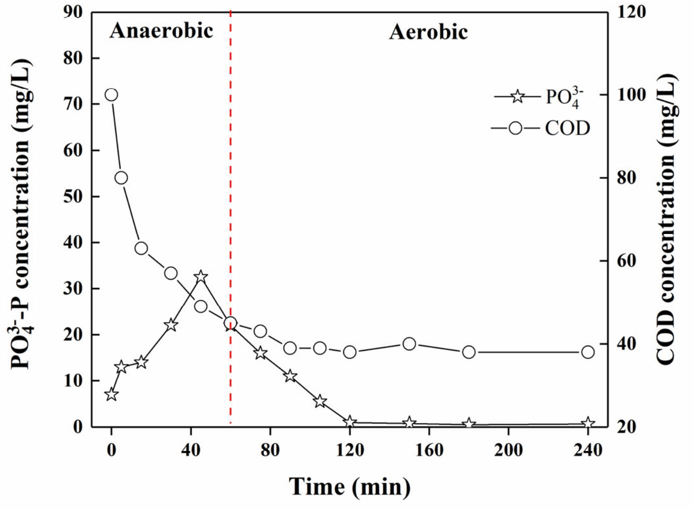

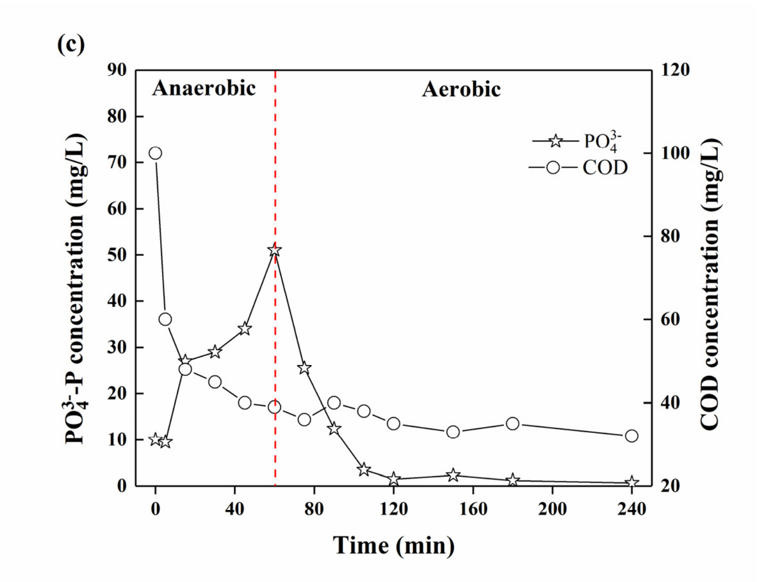

Figure S5. Profiles of PO_4^{3-}-P and COD during P release and uptake batch tests fed with propionate in (a) Phase I, (b) Phase II, and (c) Phase III.

Table S1 Summary of COD/P and COD/N ratios in high-strength dairy and manure wastewater.

Wastewater source	COD/P	COD/N	Reference
Synthetic digested manure	25.0	10.0–20.0	[1]
Synthetic dairy processing	19.5	24.1	[2]
Digested dairy manure	27.7	6.9	[3]
Diluted digested dairy manure	99.0	6.9	[4]
Diluted dairy manure	41.6	20.6	[5]
Poultry farm	37.5–42.1	6.8–8.0	[6]
Livestock farm	23.4–51.2	8.9–19.7	This study

Table S2 Component and concentrations of other trace elements in the synthetic pre-fermented high-strength wastewater.

Component	Concentration (mg/L)
Yeast extract	8
$MgCl_2 \cdot 6H_2O$	219
$MgSO_4 \cdot 7H_2O$	14
KCl	98
$CaCl_2$	46
H_3BO_3	0.061
$Na_2MoO_4 \cdot 2H_2O$	0.031
KI	0.015

$CuSO_4 \cdot 5H_2O$	0.061
$Co(NO_3)_2 \cdot 6H_2O$	0.075
$ZnSO_4 \cdot 7H_2O$	0.3

Table S3. Average specific ammonia oxidation rate (AOR) and specific nitrite oxidation rate (NOR) in typical cycles of SBR reactor during the experiment.

Phase	AOR [mg N/(g VSS·h)]	NOR [mg N/(g VSS·h)]	Reference
I	0.51	0.61	This study
II	0.66	0.42	This study
III	0.72	0.18	This study
Other activated sludge systems	3.25-3.62	4.68-5.57	[7,8]

AOR: specific ammonia oxidation rate; NOR: specific nitrite oxidation rate.

References

1. Yuan Z., Kang D., Li G., Lee J., Han I., Wang D., Zheng P., Reid M.C., Gu A.Z., Combined Enhanced Biological Phosphorus Removal (EBPR) and Nitrite Accumulation for Treating High-strength Wastewater. *bioRxiv*. **2021**.
2. Bickers P., Bhamidimarri R., Shepherd J., Russell J., Biological phosphorus removal from a phosphorus-rich dairy processing wastewater. *Water Sci Technol*. **2003**, *48*, 43-51.
3. Sooknah R.D., Wilkie A.C., Nutrient removal by floating aquatic macrophytes cultured in anaerobically digested flushed dairy manure wastewater. *Ecol Eng*. **2004**, *22*, 27-42.
4. Wang L., Li Y., Chen P., Min M., Chen Y., Zhu J., Ruan R.R., Anaerobic digested dairy manure as a nutrient supplement for cultivation of oil-rich green microalgae Chlorella sp. *Bioresource Technol*. **2010**, *101*, 2623-8.
5. Qureshi A., Lo K.V., Mavinic D.S., Liao P.H., Koch F., Kelly H., Dairy manure treatment, digestion and nutrient recovery as a phosphate fertilizer. *Journal of Environmental Science and Health, Part B*. **2006**, *41*, 1221-35.
6. Zheng T., Li P., Ma X., Sun X., Wu C., Wang Q., Gao M., Pilot-scale experiments on multilevel contact oxidation treatment of poultry farm wastewater using saran lock carriers under different operation model. *Journal of Environmental Sciences*. **2019**, *77*, 336-45.
7. Li S., Fei X., Chi Y., Jiao X., Wang L., Integrated temperature and DO effect on the lab scale A2O process: performance, kinetics and microbial community. *International Biodeterioration & Biodegradation*. **2018**, *133*, 170-9.
8. Yao Q., Peng D.-C., Nitrite oxidizing bacteria (NOB) dominating in nitrifying community in full-scale biological nutrient removal wastewater treatment plants. *Amb Express*. **2017**, *7*, 1-11.
